# First Detection of Rotavirus Group C in Asymptomatic Pigs of Smallholder Farms in East Africa

**DOI:** 10.3390/pathogens6030037

**Published:** 2017-08-14

**Authors:** Joshua Oluoch Amimo, Eunice Magoma Machuka, Edward Okoth

**Affiliations:** 1 Department of Animal Production, Faculty of Veterinary Medicine, University of Nairobi, P.O. Box 29053, Nairobi 00625, Kenya; 2 Biosciences of East and Central Africa-International Livestock Research Institute, (BecA-ILRI) Hub, P.O. Box 30709, Nairobi 00100, Kenya; e.machuka@cgiar.org (E.M.M.); e.okoth@cgiar.org (E.O.)

**Keywords:** smallholder pigs, rotavirus group C, East Africa

## Abstract

Group C rotavirus (RVC) has been described to be a causative agent of gastroenteritis in humans and animals including pigs, cows, and dogs. Fecal samples collected from asymptomatic pigs in smallholder swine farms in Kenya and Uganda were screened for the presence of group C rotaviruses (RVC) using a reverse transcription-polymerase chain reaction assay. A total of 446 samples were tested and 37 were positive (8.3%). A significantly larger (*p* < 0.05) number of RVC-positive samples was detected in groups of older pigs (5–6 months) than in younger piglets (1–2 months). There were no significant differences in the RVC detection rate between the pigs that were full time housed/tethered and those that were free range combined with housing/tethering. After compiling these data with diagnostic results for group A rotaviruses (RVA), 13 RVC-positive samples were also positive for RVA. This study provides the first evidence that porcine group C rotavirus may be detected frequently in asymptomatic piglets (aged < 1–6 months) in East Africa. The occurrence of RVC in mixed infections with RVA and other enteric pathogens requires further research to investigate the pathogenic potential of RVC in pigs.

## 1. Introduction

Smallholder pig producers own the bulk of the pigs (>80%) in sub-Saharan Africa, and present the strongest influence to the swine industry in this region. These smallholder farms are characterized by low productivity compared to larger scale commercial farms in developed countries. The major constraints hampering the growth and development of the pig industry in the region is the high disease burden. In developing countries, one of the major causes of mortality among children (<5 years) and young piglets is diarrheal disease [1,2,3]. In the swine industry, piglets that suffer from Rotavirus RV diarrheal disease have stunted growth and some may die, resulting in high economic losses. Knowledge on RV prevalence in animals in East Africa is limited, with no information on non-group A rotaviruses, but it is suspected to be among the major causes of suckling and weaning piglet diarrheal disease and a concern for pork producers due to the resulting mortality, reduced growth, and high costs of treatment and control. Limited epidemiologic surveys globally suggest that group RVCs are widespread and enzootic in pig herds [4,5]. Outbreaks of diarrhea associated with RVCs have been documented in nursing, weaning, and post-weaning pigs [6,7,8,9], either alone or in mixed infections with other enteric pathogens. Collection of epidemiological and molecular data on RVCs in animals is crucial for a better understanding of their ecology, genetic/antigenic diversity, and zoonotic potential. Evidence for the zoonotic potential of porcine RVCs was revealed by analyses of archive fecal samples of children infected with porcine-like RVCs [10]. Detection of animal-like RVCs in humans requires detailed studies of the epidemiology and genetic diversity of animal RVCs, especially in regions where humans and animals or different animal species often live in close contact, making mixed infections more common. In this study, we investigated the presence of RVCs in smallholder swine herds in East Africa, where a total of 446 fecal samples from piglets aged 1 to 6 months were screened by using RVC-specific primers based on the VP6 gene described by Amimo et al. [6].

## 2. Results and Discussion

The information on the prevalence of porcine RVCs is very limited, especially in Africa, with no historic or current reports available, apart from the reports by Geyer et al. published nearly 30 years ago [11]. The absence of surveillance programs and appropriate diagnostic facilities for porcine RVC have resulted in a lack of data on its prevalence. In this study, we found an overall prevalence of RVC of 8.3% in 446 fecal samples (37/446) from western Kenya and eastern Uganda. Of the 37 samples which were positive, 35% (13/37) of them occurred in mixed infection with RVA. The results of RVA detection was discussed in detail by Amimo and colleagues in 2015 [12]. RVC was reported both in Uganda (7.7%) and Kenya (8.8%) in nearly equal proportions. The virus strain was also present in all the sub-counties studied; with Tororo sub-county (11.5%) having a higher detection rate and Busia sub-county (5.0%) in Uganda having the lowest rate (Table 1). Tororo region is characterized by a major town in the eastern part of Uganda with a high density of pigs compared to Busia area in Uganda, which could explain the high prevalence of RVC in Tororo. Based on the age group (≤2 months, 3 and 4 months, 5 and 6 months old), RVC prevalence was higher (11.6%) in the older pigs (5 and 6 months) than the nursing piglets (4.3%), as shown in Table 1. We also examined whether the management practices had effect on the RVC detection rate; we found that the difference was not statistically significant (*p* > 0.05), however, the prevalence was slightly higher in the piglets that were free range combined with tethering or housing (9.6%) compared with the piglets that were fully tethered or housed (7.3%). The study examined the effect of the herd size on the prevalence of RVC, and found that piglets in a herd of 6–10 pigs (10.8%) were more affected. Based on the month of sample collection, there was high prevalence of RVC in July (11.8%) and November (10.5%), however, there was no evidence of seasonal influence on RVC prevalence in the study region, since July is in the dry season and November is in the short rainy season. Nonetheless, we recommend a comprehensive study to elucidate the seasonal influence on RVC prevalence in the study area.

The prevalence reported here was lower than the 19.5% reported in the USA [6], 26.3% reported in S. Korea (Jeong et al., 2009), and 28.7% reported in Italy (Martella et al., 2007). However, the previous studies in Korea and Italy analyzed only samples from diarrheic pigs, whereas we used samples from non-diarrheic pigs. The study by Amimo and colleagues in the USA examined fecal samples from both symptomatic and asymptomatic piglets, and reported an overall porcine RVC prevalence of 19.5% with 8.5% in asymptomatic weaned pigs in USA, which is consistent with our results. The study reported high RVC prevalence (23.5%) in younger piglets (<30 days old). Similarly, Marthaler and colleagues reported 46% (53% using RT-qPCR) prevalence of RVC in 7520 pig fecal samples from the USA and Canada, and their results revealed that single RVC infection was very high (78%) in neonatal piglets (<3 days) and young piglets of 4–20 days (65%) compared to older pigs of 455 days (13%) [8,9]. In Brazil, Molinari et al. [13] also reported porcine RVC to be the most prevalent group in swine herd during a post-weaning diarrhea outbreak, occurring in single (34%) and mixed (44%) infections. In China, Peng et al. [14] also reported 16.65% prevalence of RCV in 793 fecal samples from diarrheic and non-diarrheic pigs from Lulong area. Porcine RVC has been also reported in pigs in Czech Republic (4.4%), Belgium (29%), and Germany (31%) [15,16,17].

Even though we sampled piglets older than 21 days, our results indicate that porcine RVC could be an important enteric pathogen in the study region, since many studies have described porcine RVC as an important enteric pathogen in nursing and weaned piglets. Further studies are therefore required to ascertain the pathogenic potential of RVC in the swine population in East Africa to be able to develop effective measures for the prevention and control of rotavirus infections in swine.

## 3. Materials and Methods

### 3.1. Fecal Sample Collection

A total of 446 fecal samples were collected from individual piglets aged 1–6 months from two sub-counties (Busia and Teso) in western Kenya and two sub-counties (Tororo and Busia) in eastern Uganda in the year 2012 (July to November) and February, 2013. In the study region, there are three seasons based on the rainfall pattern i.e., the long rainy season (March to May), the short rainy season (September to November), and the dry season (December to February, June to August). The detailed sampling procedure has been described by Amimo et al., 2014 and 2015 [12,18].

### 3.2. RNA Extraction and Detection of Rotavirus Group C

RNA was extracted from 250 μL of 10% (*w*/*v*) fecal suspensions in phosphate-buffered saline (PBS) using an RNeasy mini kit (Qiagen, CA, USA) according to the manufacturer’s instructions. The total RNA recovered was suspended in 40 µL of nuclease free water and stored at −70 °C until used. Conventional RT-PCR was used for the detection of the RVCs with validated primer sets designed from the VP6 gene (giving 260bp PCR product), as described previously by Amimo et al. [6]. The RT-PCR assay was conducted using a one-step Qiagen RT-PCR kit (Qiagen, Valencia, CA, USA) according to the manufacturer’s instructions, with minor modifications. The amplicons were analyzed in 2% agarose gel electrophoresis and visualized by ultraviolet illumination after staining with gel red™ nucleic acid gel stain (Biotium, Hayward, CA, USA). Cell-cultured porcine RVC prototype Cowden was used as positive control in all PCR reactions. A chi-square test was used to assess the relationship between several factors (country of origin, sub-counties, age group, management systems, and herd size) and the RVC detection using procedure frequency in the SAS computer program (SAS, 2002).

## Figures and Tables

**Table 1:** Relative distribution of RVC in asymptomatic pigs in smallholder swine farms in East Africa during the study period.

Details	Groups	*N*	Positive	Prevalence (%)
	Overall	446	37	8.3
Country	Kenya	239	21	8.8
	Uganda	207	16	7.7
Sub-Counties	Busia Kenya	113	9	8.0
	Teso, Kenya	126	12	9.5
	Busia Uganda	120	6	5.0
	Tororo Uganda	87	10	11.5
Age Group	Nursing (1 ≤ 3 months)	47	2	4.3
	Weaner (3 and 4 months)	210	13	6.2
	Weaner (5 and 6 months)	189	22	11.6
Management Systems	Free range with tethering/housing	198	19	9.6
	Full-time housing/tethering	248	18	7.3
Herd Size	<6 Pigs	386	32	8.3
	6–10 Pigs	37	4	10.8
	>10 Pigs	23	1	4.3
Month of Collection	July 2012	51	6	11.8
	August 2012	134	12	9.0
	September 2012	22	0	0.0
	October 2012	39	1	2.6
	November 2012	153	16	10.5
	February 2013	47	2	4.3
